# Impact of Vein of Marshall Ethanol Infusion Combined with Anatomical Ablation for the Treatment of Persistent Atrial Fibrillation: A Long-Term Follow-Up Based on Implantable Loop Recorders

**DOI:** 10.3390/jcm12216916

**Published:** 2023-11-03

**Authors:** Martina Nesti, Fabiana Luca, Luca Panchetti, Silvia Garibaldi, Umberto Startari, Gianluca Mirizzi, Federico Landra, Alberto Giannoni, Marcello Piacenti, Andrea Rossi

**Affiliations:** 1 Fondazione Toscana Gabriele Monasterio, 56124 Pisa, Italy; martina.nesti@tiscali.it (M.N.); sgaribaldi@ftgm.it (S.G.); umberto.startari@ftgm.it (U.S.); gmirizzi@ftgm.it (G.M.); alberto.giannoni@santannapisa.it (A.G.); andrea.rossi@ftgm.it (A.R.); 2 Cardiology Department, Grande Ospedale Metropolitano, 89124 Reggio Calabria, Italy; 3 Division of Cardiology, Department of Medical Biotechnologies, University of Siena, 53100 Siena, Italy; f.landra@student.unisi.it; 4 Health Science Interdisciplinary Center, Scuola Superiore Sant’Anna, 56127 Pisa, Italy

**Keywords:** catheter ablation (CA), persistent atrial fibrillation (PeAF), vein of Marshall (VOM), VOM ethanol infusion (EI), pulmonary vein isolation (PVI), left atrial (LA) roofline, mitral line, cavotricuspid isthmus line, implantable loop recorder (ILR)

## Abstract

Background: The best ablation treatment for persistent atrial fibrillation (PeAF) patients is still debated. The vein of Marshall (VOM) seems to be a promising target for ablation and could be combined with a linear set of ablation lesions. The aim of our study is to evaluate the incidence of AF recurrences in a PeAF population treated with a comprehensive ablation approach consisting of VOM ethanol infusion (EI), pulmonary vein isolation (PVI), a left atrial (LA) roofline, a mitral line (guided by the newly formed lesion after alcohol infusion into the VOM and validated by pacing), and a cavotricuspid isthmus line. Methods: Consecutive patients undergoing the first ablation procedure of catheter ablation (CA) for PeAF were enrolled. All patients underwent VOM-EI, PVI, and ablation lines along the roof of the LA, mitral, and cavotricuspid isthmus. LA voltage mapping before and after VOM-EI was also performed. An implantable loop recorder (ILR) was implanted at the end of the ablation in each patient. Results: Thirty-one consecutive patients (66 ± 8 years and 71% male) affected by PeAF were included in this study. The VOM-EI procedural phase lasted 21.4 ± 10.1 min. PV isolation and lines were validated in all subjects. The ML block was achieved within 10.8 ± 8.7 min. At a mean follow-up of 12 ± 7 months, 27 out of 31 (87%) patients remained free from AT/AF recurrences. Among the patients with recurrences, two (50%) had incomplete ablation lesions and three (75%) had “suboptimal” VOM-EI. In 23/31 patients (74%), antiarrhythmic drugs (AADs) were discontinued after 1 month of follow-up. No significant complications were reported during the follow-up. Conclusions: this single-center experience demonstrates that VOM-EI systematically combined with an anatomical ablation set in patients with PeAF resulted in feasible, safe, and effective freedom from AF/AT recurrences in 87% of the population after a 1-year follow-up period according to an ILR.

## 1. Introduction

Catheter ablation (CA) is a cornerstone of treatment for atrial fibrillation (AF), and pulmonary vein isolation (PVI) is a proven, effective approach for paroxysmal AF ablation [1,2,3,4]. However, procedural outcomes in patients with persistent AF (PeAF) remain modest [5,6,7,8]. In these patients, additional lesions beyond PV isolation have failed to improve outcomes in prospective randomized trials [9].

The vein of Marshall (VOM) is an embryological remnant from the left superior vena cava and appears to play a key role in atrial arrhythmogenesis. Atrial myocardial tissue surrounding the vein of Marshall (VOM) has been shown to be able to sustain focused electrical activity [10] and stable reentries [11] for priming AF and has demonstrated to be correlated with the presence of autonomic parasympathetic [12,13] and sympathetic [14] innervation [15]. Due to its perfect fit with Coumel’s triangle’s components (trigger, substrate, and autonomic tone), the VOM is a promising therapeutic target.

Targeting the VOM with chemical ablation is also a promising and effective method of facilitating and reinforcing the lateral mitral line (ML), a critical zone of common ablation strategies in PeAF and perimitral flutters [16]. Indeed, alcohol infusion into the VOM facilitates a bidirectional block across the line, eliminating protected epicardial connections. Recent studies suggest that VOM ethanol infusion (VOM-EI) combined with PVI may decrease the risk of AF recurrence in PeAF patients [17,18], but the researchers performed coronary sinus and great cardiac vein (GCV) radiofrequency ablation, targeting epicardial muscular bundles for all patients. In our study, additional radiofrequency ablations were performed at the GCV only in presence of residual epicardial connection across the mitral line (ML) to reduce the procedural time and increase the safety of procedure. Notably, in these studies, an implantable loop recorder (ILR) was not used to reliably exclude the risk of AF recurrences. The aim of our study was to evaluate the incidence of AF recurrences by systematically using ILPSs in a PeAF population treated with a comprehensive ablation approach encompassing VOM-EI, PVI, a roofline, a mitral line (guided by the newly formed lesion after alcohol infusion into the VOM and validated by pacing), and a cavotricuspid isthmus line. Moreover, the use of a loop recorder allows for episodes of silent AF recurrences to be detected in order to clearly understand the real impact of VOM-EI in AF treatment.

## 2. Methods

### 2.1. Patient Selection

Consecutive patients undergoing the first ablation procedure of CA for PeAF at Fondazione Toscana Gabriele Monasterio (Pisa, Italy) from April 2021 to January 2023 were considered. Patients with an identifiable VOM were prospectively included in this study, and data were collected on a dedicated database. Patients were excluded if they were under 18 years of age or if they did not provide informed consent. All procedures were performed under general anesthesia. This study was performed in accordance with the Declaration of Helsinki and reviewed and approved by our institutional review board.

### 2.2. Voltage Mapping

Electrophysiological procedures were performed using either CARTO 3 ^®^ (Biosense Webster, Inc., Bar, CA, USA) or EnSite X ^®^ (Abbott, Inc., Lake Bluff, IL, USA) 3D mapping systems. High-density bipolar voltage mapping at the MI level with either PentaRay TM or Advisor HD Grid TM catheters was performed before and immediately after VOM-EI. Bipolar map cutoffs were set to 0.05–0.50 mV in case of sinus rhythm or 0.05–0.29 mV in case of atrial fibrillation.

### 2.3. Vein of Marshall Ethanol Infusion

Ethanol infusion into the vein of Marshall was performed before catheter ablation. Coronary sinus cannulation was achieved with either non-steerable (Swartz SL0, Abbott, Inc.) or steerable sheaths (Agilis NxT, Abbott, Inc.). The VOM was initially localized via coronary sinus venography and thereafter selectively cannulated using a left internal mammary artery diagnostic catheter. A 0.014” guidewire and an over-the-wire (OTW) angioplasty balloon (1.5–2–2.5 mm × 8–12 mm) were used to occlude the VOM at its ostium. Additional VOM venography throughout the OTW balloon was performed to confirm its position. VOM-EI was then performed: a total of 10 cc of ethanol was infused for 10 min in bolus of 3 or 4 cc, and supplementary boluses were infused in case of overt leakage. After each bolus of ethanol, 1 cc of medium contrast was injected to check the leakage, myocardial contrast staining, and eventual VOM perforation/dissection with contrast medium extravasation in the pericardial space. Intracardiac echocardiography was used to monitor the procedure. At the end of procedure, we defined “suboptimal VOM-EI” as the lack of stability of the balloon with recoil and no proximal VOM occlusion, a significant leakage in the coronary sinus that was not correctable, and the absence of overt myocardial contrast staining after the total dose of alcohol was infused. The ablation was considered “incomplete” when ML block was not achieved after application of radiofrequency at the GCV level.

### 2.4. Catheter Ablation

CA was subsequently performed with the use of radiofrequency energy delivered by QDOT MICRO TM (Biosense Webster, Inc.) or TactiCath TM (Abbott, Inc.) ablation catheters. Ablation setup started with wide antral PVI and subsequently proceeded with linear lesion for the LA dome (roofline), lateral ML, and cavotricuspid isthmus. In the case of AF persistence after PVI, an external cardioversion was performed. Ablation lines were performed with point-by-point radiofrequency delivered with an interlesion distance of 4 mm for 15–20 s with a power output of 40–45 W, a temperature limited to 45 degrees, and normal saline irrigation (8–20 mL/min). Roofline and ML block were subsequently confirmed during left atrial appendage pacing at 600 msec cycle length. In the presence of residual epicardial connection across the mitral line (ML), additional radiofrequency ablations were performed at the great cardiac vein (GCV), either at the anchoring or free-wall side, for 20 s with a power output of 20 W. Immediately after the procedure, all patients underwent ILR implantation.

### 2.5. Follow-up

**Figure 1:**
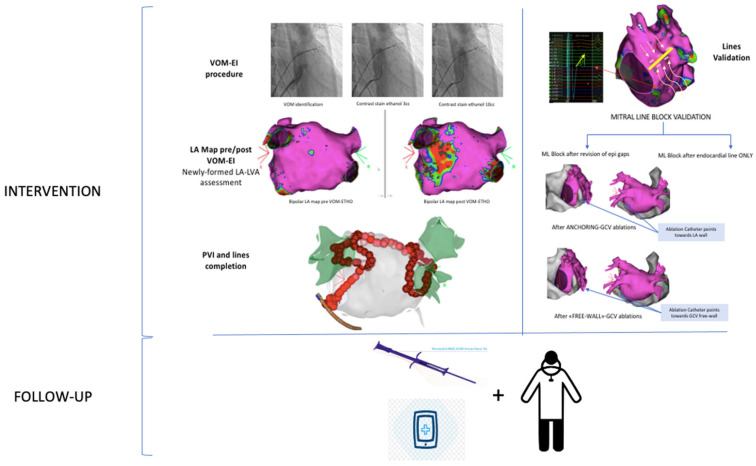
Freedom from AT/AF recurrences.

Cardiac rhythm was continuously monitored using a loop recorder (Reveal Linq^®^; Medtronic, Minneapolis, MN, USA) implanted just after ablation treatment. These devices are equipped with an AF detection algorithm based on beat-to-beat variability, resulting in accurate detection of AF episodes and AF burden (percentage of time in AF) [19]. All patients were followed up with a 12-lead electrocardiogram, 24 h Holter recording, transthoracic echocardiogram at 1, 6, and 12 months and remote monitoring. Recurrence was defined as any episode of AT/AF lasting >30 s after a 1-month blanking period post-ablation, verified by a blinded board cardiologist (Figure 1). Procedure-related complications, such as cardiac tamponade, delayed pericarditis, vascular damage, and atrial-esophageal fistula, were reported. The study protocol is described in Figure 1.

### 2.6. Statistical Analysis

Continuous variables were reported as mean ± SD or as median and interquartile ranges, according to variable distribution, while categorical variables were reported as absolute numbers (percentage). Kaplan–Meier analysis was used to describe the clinical outcome represented by arrhythmic recurrence. *p* < 0.05 was considered significant.

## 3. Results

### 3.1. Population

Thirty-one consecutive patients affected by PeAF were included in this study, fifteen of whom had long-standing AF. Baseline characteristics are reported in Table 1. The mean age was 66 ± 8 years, and 22/31 (71%) patients were male. Thirteen patients had overt structural heart disease, mainly ischemic (19.4%) and AF-induced (19.4%) cardiomyopathy. The mean CHA2DS2-VASc score was 2.48 ± 1.48. Almost all of the population was taking AADs, mainly amiodarone and class Ic drugs. The mean duration of AF episodes was 8.1 ± 4.4 months, and 12/31 (38.7%) subjects presented AF at the start of the procedure.

Procedural data are listed in Table 2.

### 3.2. Vein of Marshall Ethanol Infusion

VOM-EI was performed for all patients. The volume of ethanol infused was 10.7 ± 2.0 mL. The VOM-EI procedural phase lasted 21.4 ± 10.1 min with a fluoroscopy time of 10.1 ± 3.9 min and a radiologic exposure of 2454.5 ± 112.2 cGy/cm^2^. “Suboptimal VOM-EI” occurred in three patients. Complications occurred in 5/31 (16.1%) cases: 2 patients had VOM dissection and 3 had VOM perforation with contrast medium extravasation in the pericardial space. In two cases, mild pericardial effusion occurred in the absence of hemodynamic instability with subsequent spontaneous resolution. Two patients had acute pericarditis treated with anti-inflammatory drugs and colchicine.

### 3.3. LA Mapping

Thirteen patients (41.9%) showed low-voltage areas in the left atrium (LA-LVAs) on the basal map. After VOM-EI, we observed significant newly formed lesions (8.2 ± 4.4 cm^2^) throughout VOM trajectory, mainly in the area of the lateral mitral isthmus towards the left pulmonary inferior veins (which are often involved and already electrically isolated) or towards the carina between the left inferior and left superior veins.

### 3.4. Ablation

PV isolation was achieved and validated in all subjects. The roofline was verified and validated in 28/31 (90.3%) cases, while in 3 patients, bidirectional block across the line was not achieved due to residual epicardial connections. ML block was accomplished in 30/31 (96.8%) patients within 10.8 ± 8.7 min; in 17/31 patients, bidirectional ML block was seen only after endocardial RF applications, while in 13 it was only observed after additional ablations targeting coronary sinus musculature (8 in anchored GCVs and in 5 free-walled GCVs).

### 3.5. Follow-up

**Figure 2:**
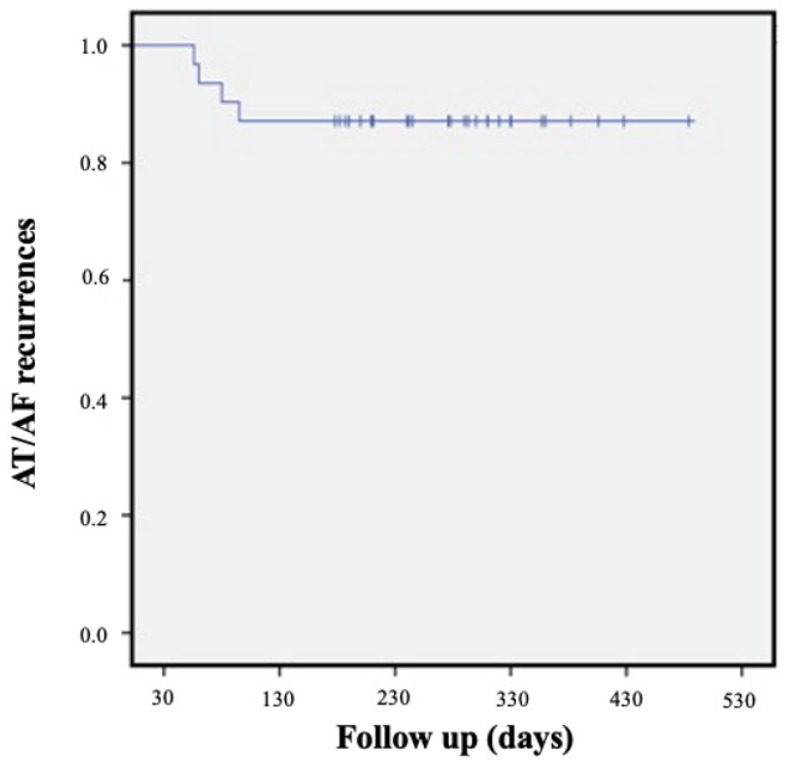
KM global population.

After a mean follow-up of 12 ± 7 months, 27 out of 31 (87%) patients remained free from AT/AF recurrences (Figure 2). Among patients with arrhythmic recurrences (*n* = four), three presented AF, while one presented AT. In this group, in two cases (50%), the ablation lesion set remained incomplete, and in three (75%), VOM-EI was considered “suboptimal”. In 23/31 patients (74%), AADs were discontinued after a 1-month follow-up. No significant complications were reported during the follow-up (Table 3 and Table 4).

## 4. Discussion

In this study, we examined a prospective population of consecutive patients with PeAF treated with VOM-EI systematically in combination with a comprehensive anatomical RF-ablation approach (wide antral PVI, roofline, lateral mitral line and cavotricuspid isthmus line). The main findings of this study are that VOM-EI is (i) feasible (global procedural duration 21.3 ± 10.1 min) and safe; (ii) effective even in patients with PeAF, considering that 87% of patients remained free from AT/AF recurrences at the implantable LRI after a 12 ± 7-month follow-up; (iii) has a low arrhythmic recurrence rate despite AAD discontinuation in most patients (23/31, 74%); and (iv) less effective, mainly in those cases in which the anatomic ablation set remained incomplete or VOM-EI was “suboptimal”.

The sustaining mechanisms underlying PeAF remain a debated topic, and evidence to support any adjunctive ablation strategies is lacking. Numerous anatomical structures have been suggested as non-pulmonary vein triggers, but their clinical utility is still unclear [20].

Randomized trials demonstrated that additional ablation lines (along the left atrial roof and the MI), complex fractionated electrograms ablation, or posterior wall isolation did not result in better outcomes in patients with PeAF [9,21]. Indeed, in a meta-analysis of 17 studies with different settings for PeAF ablation, the rate of single-procedure freedom from AF at 12 months was only 61.9% [22]. The difference between the literature and our results can be explained by the systematic approach used in our setting: the ML was accomplished through VOM-EI and, if the block was not achieved, it was completed with endocardial and epicardial ablation. The efficacy of VOM-EI in maintaining sinus rhythm was previously described in randomized trials [17,18], but in VENUS-AF [17], a higher incidence of relapses was described, probably due to the different patient population (i.e., patients with larger atrial volumes), suggesting the importance of patient selection in evaluating patients with PeAF.

Furthermore, the role of VOM-EI as the first step in PeAF ablation has rarely been investigated. Recently, Gilles et al., in a randomized study, demonstrated that VOM-EI performed as a first step before radiofrequency ablation markedly facilitates achievement of ML block and reduces the number of touch-up applications needed compared with radiofrequency ablation as a first step, even if the sequence of ablation steps did not affect the final incidence of block [23].

Also, data obtained in a recent meta-analysis confirmed that adjunctive VOM-EI is more effective than the conventional approach without increased risk [24]. VOM-EI was defined as an independent predictor of relapse [25] of atrial tachycardia [26]. A relevant point of strength in our study was the use of an LRI for all patients during follow-up in order to detect asymptomatic AF episodes. Derval et al. [18] had an incidence of recurrences similar to ours but without continuous monitoring. On the other hand, in the VENUS trial [17], only about half of patients had an implanted device to detect recurrence. The use of loop recorders was very relevant in particular in this first phase of research investigating the efficacy of VOM-EI and allowed us to obtain important results with a low incidence of complications. The most common complications of implantation are pain at the implant site and a local pocket infection or local skin reaction that could be treated via removal of the device, as well as weak R-wave sensing, which may require moving the device to another location [27]. In our population, we did not observe any kind of complication related to loop recorder implantation. The use of continuous monitoring was also described in the CIRCA-DOSE study to detect the burden of AF after cryoballoon ablation and contact force–guided radiofrequency ablation [28]. This allows the results of these two VOM-EI techniques to be compared even though the population of the CIRCA-DOSE study is represented by paroxysmal AF refractory to drug therapy. The data support the high efficacy of adding VOM-EI to standard ablation (freedom from arrhythmic recurrences at 12 months was 53.9% for radiofrequency ablation, 52.2% for cryoablation with lesions performed in 4 min, and 51.7% for cryoablation with lesions performed in 2 min).

The mechanism of improved rhythm control achieved with VOM-EI is multifactorial and includes, beyond the elimination of AF triggers and denervation, a more reliable conduction block at the MI.

Therefore, the validation of ablation lines and the effectiveness of VOM-EI is crucial. Among our patients, four had AT/AF recurrences, two had an incomplete lesion set, and three had “suboptimal” VOM-EI. Incomplete MI block seems to be the most significant cause of clinical failures of ablation, supporting the efficacy of VOM-EI in achieving this goal faster. Also, the significant newly formed lesions across the VOM trajectory observed in LA voltage mapping after VOM-EI (8.2 ± 4.4 cm^2^ vs. 2.3 ± 3.5 cm^2^) represent an index of the effectiveness of the procedure in achieving MI block.

This result was also confirmed by Yewei et al. in a cohort of 191 patients who had undergone PVI and bidirectional block in the roofline, cavotricuspid isthmus, and MI with or without VOM-EI. In the first group, MI block was achieved in 63 (95.5%), while in the second, it was achieved in 101 (80.8%) [29].

The complications observed during VOM-EI were mainly due to perforation in the dissection of the VOM and may have been related to the learning curve of the VOM-EI procedure in our laboratory. Still, they did not result in significant complications during the procedure or subsequent follow-up. Our procedural time was similar to previously described experiences [17,22], and overall, we confirmed that VOM-EI is a feasible and safe procedure that can be performed in a short time.

### Limitations

This study has some important limitations: it describes the experience of a single center without a group of controls. Further multicenter studies are warranted to define the role of this ablation setting in the outcome of PeAF patients, in particular compared with other strategies. Moreover, antiarrhythmic drug discontinuation, which is not always due to medical decisions, can influence the incidence of recurrences, and a stricter drug regimen is recommended to analyze the real efficacy of the technique.

## 5. Conclusions

This single-center experience demonstrates that vein of Marshall ethanol infusion systematically combined with anatomical ablation in patients with PeAF resulted in a feasible, safe, and effective treatment with freedom from AF/AT recurrences in 87% of the population after a 1-year follow-up period. Our results were validated by using, for the first time, continuous remote monitoring after systematic LRI at the time of the procedure. Further multicenter studies with a randomized design and longer follow-up periods are warranted to define the role of this ablation setting in the outcome of PeAF patients.

## Figures and Tables

**Table 1:** Baseline characteristics.

	Patients (*n* = 31)
Age (yr)	66 ± 8
Male sex, *n* (%)	71
Structural cardiac disease, *n* (%) Ischaemic Valvular heart disease Hypertrophic cardiomyopathy Dilated cardiomyopaty AF-induced cardiomyopathy Previous open-chest surgery	13 (58.1) 6 (19.4) 2 (3.2) 2 (6.5) 1 (2.1) 6 (19.4) 2 (6.5)
Hypertension (%)	71
CHA2DS2-VASc score (mean)	2.48 ± 1.48
**Antiarrhythmic Drugs *n* (%)** Amiodarone Ic drugs Sotalol	30 (96.8) 16 (51.6) 11 (35.5) 3 (9.7)
**AF Characteristics**	
Maximum duration (mo)	8.13 ± 4.37
Long-standing AF, *n* (%)	15 (48.4)
AT/AF at the start of the procedure, *n* (%)	12 (38.7)
**Echocardiographic Parameters**	
Left atrial volume (mL/m^2^)	32 ± 5.00
Telediastolic ventricular diameter (mm)	50 ± 3.63
Left ventricular ejection fraction (%)	56 ± 11.22

**Table 2:** Procedural data.

	Patients (*n* = 31)
**VOM Ethanol Infusion**	
VOM-EI time (min)	21.29 ± 10.07
VOM-EI fluoroscopy time (min)	10.13 ± 3.91
Ethanol infused (cc)	10.71 ± 2.00 (8–16)
**Complications Overall, *n* (%)** VOM dissection VOM perforation Pericardial effusion Tamponade	5 (16.1) 1 (3.2) 3 (9.7) 2 (6.5) 0
Incomplete ethanol delivery	4 (12.9)
Acute pericarditis	2 (6.5)
Ethanol infusion in wrong veins	0
Stroke	0
Vascular complications	0
**Left Atrium Voltage Analysis**	
Presence of left atrium low-voltage areas, *n* (%)	13 (41.9)
Basal LA-LVA (cm^2^)	2.30 ± 3.45 (0–14)
Newly formed LVA post-VOM-EI (cm^2^)	8.22 ± 4.40 (0–16.50)
**Ablation**	
PVI completion/validation, *n* (%)	31 (100)
Roofline block, *n* (%)	28 (90.3)
Mitral line block, *n* (%) Endocardial only RF anchoring—GCV RF free wall—GCV	30 (96.8) 17 (54.8) 8 (25.8) 5 (16.1)
Cavotricuspid isthmus block, *n* (%)	31 (100)
Anatomical lesion set completed, *n* (%)	27 (87.1)
Time to mitral line block (min)	10.77 ± 8.72 (2–36)

**Table 3:** Follow-up data.

Mean duration (months)	12 ± 7
AT/AF recurrences, *n* (%)	4/27 (87%)
AAD discontinuation, *n* (%)	23/31 (74%)
Delayed pericarditis, *n*	0
Delayed tamponade, *n*	0

**Table 4:** Details of Patients with AT/AF Recurrences.

	(*n* = 4)
**Type of Recurrence**	
Recurrence as AT, *n* (%)	1 (25%)
Recurrence as AF, *n* (%)	3 (75%)
**Lesion Set**	
Completed lesion set, *n* (%)	2 (50%)
Roofline block, *n* (%)	1 (25%)
Mitral line block, *n* (%)	1 (25%)
Suboptimal VOM-EI, *n* (%)	3 (75%)

## Data Availability

Not applicable.
